# Background matching and disruptive coloration as habitat-specific strategies for camouflage

**DOI:** 10.1038/s41598-019-44349-2

**Published:** 2019-05-24

**Authors:** Natasha Price, Samuel Green, Jolyon Troscianko, Tom Tregenza, Martin Stevens

**Affiliations:** Centre for Ecology and Conservation, College of Life and Environmental Sciences, University of Exeter, Penryn Campus, Penryn, TR10 9FE UK

**Keywords:** Behavioural ecology, Evolutionary ecology

## Abstract

Camouflage is a key defence across taxa and frequently critical to survival. A common strategy is background matching, resembling the colour and pattern of the environment. This approach, however, may be ineffective in complex habitats where matching one patch may lead to increased visibility in other patches. In contrast, disruptive coloration, which disguises body outlines, may be effective against complex backgrounds. These ideas have rarely been tested and previous work focuses on artificial systems. Here, we test the camouflage strategies of the shore crab (*Carcinus maenas*) in two habitats, being a species that is highly variable, capable of plastic changes in appearance, and lives in multiple environments. Using predator (bird and fish) vision modelling and image analysis, we quantified background matching and disruption in crabs from rock pools and mudflats, predicting that disruption would dominate in visually complex rock pools but background matching in more uniform mudflats. As expected, rock pool individuals had significantly higher edge disruption than mudflat crabs, whereas mudflat crabs more closely matched the substrate than rock pool crabs for colour, luminance, and pattern. Our study demonstrates facultative expression of camouflage strategies dependent on the visual environment, with implications for the evolution and interrelatedness of defensive strategies.

## Introduction

Many animals exhibit visual similarities with their environment, commonly referred to as ‘phenotype-environment associations’^1,2^. It has long been appreciated that these associations are a product of natural selection^3,4^, with individuals camouflaged against the prevailing visual environment^5^. Indeed, selection has driven correlations in appearance between individual phenotypes and backgrounds in a wide range of animals, from rodents and lizards in terrestrial habitats^6,7^ to crabs in marine habitats^2,8,9^, and even in plants^10^. These examples of phenotype-environment associations are highly suggestive of a camouflage function, but past work has rarely demonstrated or quantified the actual camouflage resulting from any match to the local environment; that is, phenotype-environment matching (but see^1^). Nonetheless, camouflage, and its effectiveness, is widely appreciated to involve an interaction between the appearance of the organism and that of its background. This poses the question of how camouflage should be tuned to work best in different visual environments and contexts. Indeed, a great deal of work in artificial systems has explored how camouflage can be optimised under specific contexts (see below).

Phenotype-environment associations have to date largely been considered in the context of background matching^11,12^, a widespread form of camouflage involving resembling the general colour and pattern of the environment (e.g.^13^). Background matching reduces the deviation in features between the appearance of an animal and its surroundings, and is therefore specific to the visual background where it has arisen^12^. While not always directly quantified in animals, background matching is likely to be widespread across many habitats and species, ranging from classic examples of concealment (e.g.^14,15^) to studies demonstrating its utility in preventing detection of potential prey in both the lab (e.g.^16^) and field^13^. At least one study^1^ has also provided direct quantification of site-specific background matching in an intertidal crustacean, the sand flea (*Hippa testudinaria*), demonstrating that individuals match the colour and luminance of their own beaches more closely than of neighbouring beaches.

The efficacy of background matching can be limited by the outline of an animal’s body, creating discontinuities with the background that make it more conspicuous to predators^17^. As a solution, disruptive coloration involves relatively high contrast markings near the edge of the body to break up the outline^3,17,18^. Considerable research on disruptive coloration has demonstrated how this works, using artificial (human-made) prey presented to either birds or humans (e.g.^19–24^). These studies have shown that disruptive coloration frequently provides a reduction in detection (and potentially identification too), over and above the benefits conferred by background matching, and works at least in part by creating false edge information and hiding true body outlines^18,25^.

Disruptive coloration has been suggested as a key method of camouflage across numerous taxonomic groups (see review^26^). However, studies have seldom quantified the camouflage effect of disruptive coloration in real animals, or even clearly demonstrated its presence. Work has been overwhelmingly focussed on artificial systems, or has largely subjectively inferred disruption. Merilaita^27^ analysed the distribution of spots on the polymorphic marine isopod *Idotea baltica* and found that spots deviated from a random distribution, being more likely to occur at the body edges. While consistent with disruption, this study did not compare the distribution of body markings to the actual distribution in the environment, nor directly quantify any potential disruptiveness of the markings either to vision models or in behavioural trials. A more recent study used a model of edge disruption to assess the behavioural choice of resting position in two species of moth, and found that both disruptive coloration and background matching were used to provide camouflage^28^. A similar earlier model also showed evidence for disruptive coloration and false edges in frogs^29^.

Overall, there remain several substantial gaps in our understanding of how camouflage is utilised in nature. As discussed, much past work on phenotype-environment matching has relied on human subjective judgement and does not quantify actual camouflage match among habitats; background matching is often inferred rather than directly quantified. Second, current evidence for the use of disruption in nature remains very limited and seldom quantified in any real animal. The vast majority of work is limited to artificial (human-made) systems. Third, no study has tested and compared the occurrence of both background matching and disruptive coloration strategies in an animal, especially in phenotypically variable species, nor investigated the use of these strategies across habitats. As yet, few quantitative tests exist directly comparing how camouflage types are expected to vary in use with habitat type/spatial scene, and predictions from artificial systems require testing in real animals and environments. Finally, while both background matching and disruptive coloration are effective means of preventing detection, there has been debate as to what extent these strategies operate independently^21,30–32^. Indeed, some studies suggest that maximum camouflage is achieved when both strategies are used in conjunction, and past work often shows that the benefits of disruption decline as body markings increasingly mismatch the background^30,32,33^. However, again these studies have been restricted to simple artificial prey markings and few visual backgrounds (mostly tree bark).

A valuable group to test the relative occurrence and tuning of camouflage strategies is crabs, which show great diversity in colours and patterns within and among species, including for camouflage^34,35^. In crabs, phenotype-environment associations are thought to be common with many suggested examples, often including species that can vary greatly in appearance^2,8,9,36,37^. However, these studies generally lack quantification of the match between individuals and the environment (but see^38^). Multiple studies have involved the common shore crab (*Carcinus maenas*). Early descriptive work showed associations between appearance and crab size/age and habitat^39,40^. More recent work found similar results, including showing associations between crab colour patterns in different habitats (mussel beds, rock pools, seaweed, sandy beach and rocks) at varied spatial scales^2,9,41^, including when considering predator vision^8^. In general, crabs from mudflats tend to be more uniform, greener, and with less patterning than crabs from rock pools. In addition, more uniform environments (e.g. mudflats) harbour crabs with lower intraspecific diversity than heterogeneous habitats (e.g. rock pools)^9^. Mudflat crabs are thought to use habitat-specific background matching, whereas crabs from rock pools possess high contrast and prominent markings, often found near the body edges, that are highly suggestive of disruptive coloration^2^.

**Figure 1:**
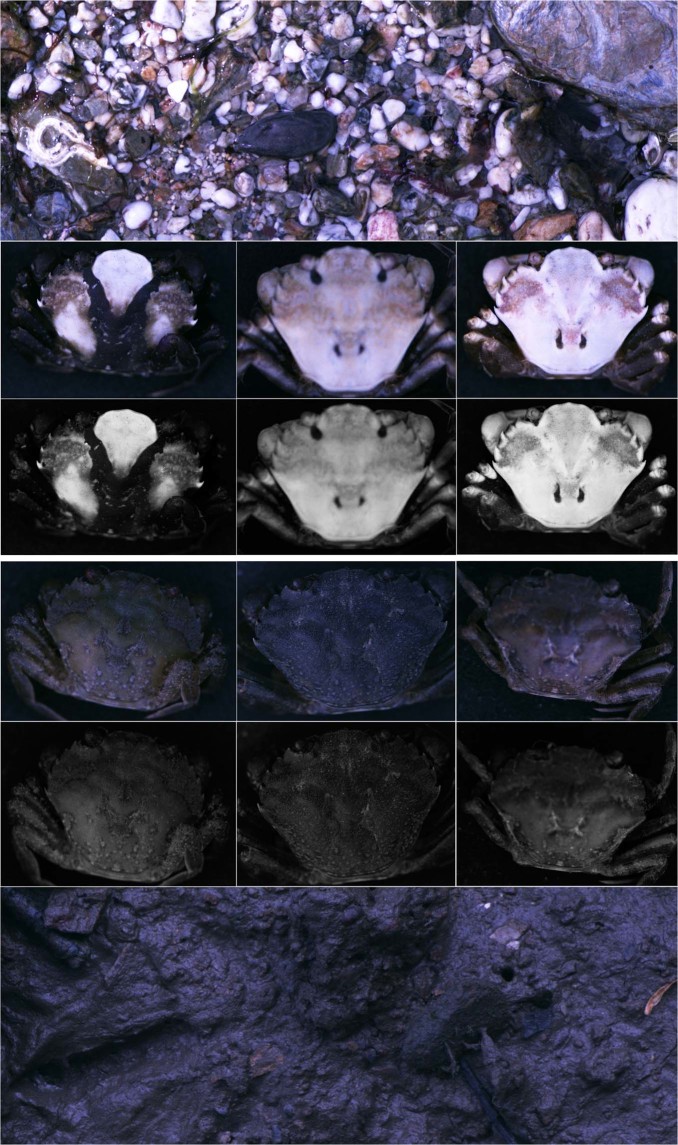
Images of a representative rock pool background (top; without crabs) and three rock pool crabs with high disruptive coloration scores. All images are converted to modelled avian vision. The top row of crab images are colour, with the red and green image layers comprising data from the LW and MW cones, and the blue layer comprising the combined data for the SW and UV cones (since images can only display three colour layers). The second row of images are luminance (lightness) images corresponding to the double cones. The images of crabs below are for individuals from mudflat habitats (colour and luminance), with an example mudflat background at the bottom.

In this study, we test for habitat-specific camouflage strategies in shore crabs across two habitat types. Using quantitative image analysis and predator (bird and fish) vision modelling (Fig. 1), we compare the match of crabs to their backgrounds for colour, luminance (perceived lightness), and pattern, and the extent of disruptive coloration using recent metrics to assess both pattern matching and edge disruption that have been demonstrated to predict visual detection and predation rates^13,23^. We predict that shore crabs from heterogeneous and contrasting rock pool habitats camouflage themselves through disruptive mechanisms, whereas crabs from uniform mudflats use background matching in their low-contrast, homogenous environment.

## Results

### Background matching: colour and luminance

**Figure 2:**
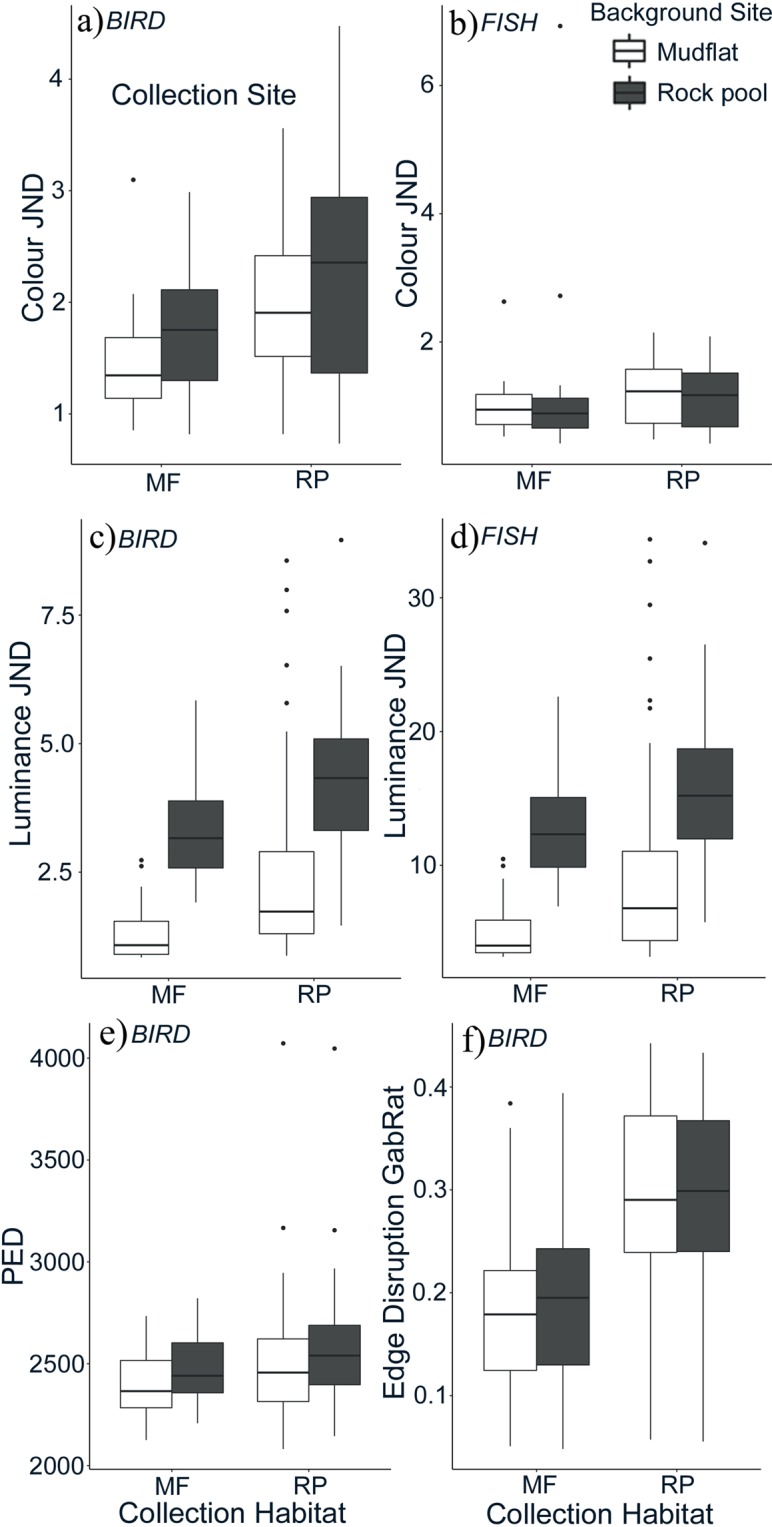
Discrimination values (JNDs) for crabs collected from either mudflat (MF) or rock pool (RP) sites (X axis) compared to either mudflat or rock pool backgrounds, corresponding to (**a**) avian colour, (**b**) fish colour, (**c**) avian luminance, and (**d**) fish luminance vision comparisons. In all cases except (**b**) crabs are a closer match to mudflat than rock pool substrates, especially when originating from mudflat backgrounds. Plots (**e** and **f**) show results from pattern analyses for background matching and disruptive coloration, respectively. For background matching, smaller pattern energy differences (PED) equate to a closer match. Here, mudflat crabs show closer matches (better camouflage) than rock pool crabs to the mudflat substrate. For disruption, larger scores (GabRat) equate to higher disruptive coloration, and here rock pool crabs show greater disruptive coloration than mudflat crabs. Boxplots show average values (bold line), interquartile range (box component), range of minimum and maximum values, and outliers (circles).

To assess how well crabs matched the colour and luminance of their backgrounds, discrimination or just noticeable difference (JND) values were used to determine the deviation between the carapace of an individual and the background (rock pool or mudflat). With the avian vision model, colour JND values were significantly predicted by an interaction between crab habitat of origin and the background habitat it was compared to (F_1,93_ = 4.09, p = 0.045, Fig. 2). This indicates that the level of background matching for colour was dependent on the habitat the individual was collected from, but the magnitude of the effect differed between habitat types. The closest match to the background (lowest JND) was seen in crabs collected from mudflats against mudflat habitat images (avian model average JND = 1.65). Conversely, the poorest match was in crabs collected from rock pools against a rock pool background (avian average JND = 2.05). Rock pool individuals on mudflat backgrounds (avian average JND = 1.76) were a marginally better match to the background than rock pool crabs on a rock pool background. Using the fish vision model we found no significant interaction between source habitat and background. The background main effect was significant (F_1,93_ = 6.11, p = 0.015). JND values were lower for fish vision relative to avian vision (see Fig. 2). Overall, however, colour matches to each background type were close (low JNDs) for all colour comparisons regardless of the specific combination of habitat type and crab habitat origin, for both avian and fish visual systems (JND average matches were all between 1.00 and 2.10). Thus, colour camouflage was good regardless of crab-habitat match.

Luminance JND between crab carapace and background was analysed using both avian and fish visual systems. There was a significant interaction between the crab habitat of origin and the background type for both visual systems (avian: F_1,93_ = 10.98, p = 0.001; fish: F_1,93_ = 8.03, p = 0.006, Fig. 2). Similar to the colour JND, the lowest luminance JND values came from shore crabs collected from mudflat sites against a mudflat background. Differences were more marked for luminance than for the colour comparisons. For avian vision, average matches of crabs to mudflat habitats were 1.79 and 1.95 JNDs for crabs from mudflats and rock pools respectively. That is compared to analogous matches of 3.59 and 3.73 JNDs for crabs against rock pool backgrounds. For fish vision, luminance average JNDs for crabs against mudflats were 6.76 and 7.35, versus matches of 13.40 and 13.92 for crabs against rock pools.

### Background matching: pattern

To assess how well crabs matched the pattern of their backgrounds, pattern energy difference (PED) values were used to determine the difference in pattern spectra between the carapace of an individual and the background habitat (rock pool or mudflat). There was a significant interaction between crab habitat and background habitat (F_1,88_ = 4.66, p = 0.033; Fig. 2). This indicates that the level of pattern background matching depended on the habitat the individual was collected from, but the magnitude of the effect differed between habitat types. The closest match to the background (lowest PED) was seen in crabs collected from mudflats and against mudflat habitat images. Conversely, the largest difference between carapace and background was in crabs collected from rock pools against a rock pool background. Otherwise, rock pool individuals on mudflat backgrounds had lower PED values and therefore were a better match to the background than rock pool crabs on a rock pool background. This shows that the phenotype of rock pool crabs is actually closer in pattern to the properties of mudflats than rock pools.

### Disruption

GabRat measurements of crab carapace disruption produced values ranging from very low (0.04) to values considered highly disruptive (>0.40)^23^. There was a significant interaction between the habitat crabs originated from and the background habitat they were compared to (interaction: F_1,84_ = 9.96; P = 0.002, Fig. 2). Overall, crabs collected from rock pools had significantly higher levels of edge disruption against both rock pool and mudflat background than crabs from mudflats. The edge disruption of crabs collected from rock pool sites was not greatly different against rock pool as opposed to mudflat backgrounds, although rock pool crabs had marginally more disruptive edges on mudflat backgrounds than they did against their own habitat substrate (Fig. 2). Conversely, mudflat crabs have slightly higher edge disruption levels on rock pools than against their own substrate background.

## Discussion

We assessed two different camouflage strategies (background matching and disruptive patterning) in juvenile shore crabs collected from rock pool and mudflat habitats. In line with our predictions, there was a clear difference in the degree of similarity of crabs to the background environment and in levels of edge disruption, depending on whether crabs were collected from rock pools or mudflats. Specifically, crabs collected from the more heterogeneous rock pools had a higher level of disruptive patterning than crabs from mudflats, and this effect was in fact marginally more pronounced against a mudflat background than against a rock pool background. This indicates that the disruptive effect operated on both backgrounds and is therefore somewhat intrinsic to the crabs themselves. Analysis of background matching found that crabs collected from the more homogeneous mudflat habitats were better matched to mudflat backgrounds than to rock pool backgrounds, and this was generally the case for colour, luminance, and pattern. Rock pool crabs compared to rock pool backgrounds often had the lowest level of background matching, whereas mudflat crabs against mudflat backgrounds had the closest overall matches. However, we note that for colour the visual modelling indicates that the level of match, and therefore camouflage, was very good for all colour comparisons regardless of habitat. Consistent with this, for colour, there was little difference in matching to a fish visual system, and only a marginally better match of crabs to the mudflat background to the avian model. In contrast, the results for luminance match were clearer than for colour, with mudflat crabs a closer match to their habitat for both visual systems. This was especially the case for the fish visual model. Overall, rock pool individuals match their habitat less closely but have high levels of edge disruption, whereas mudflat individuals do not have disruptive markings but match their background closely for colour, pattern, and luminance. These findings support our predictions that differences in camouflage strategy arise between individuals found in mudflat and rock pool habitats.

Our results build on earlier work investigating shore crab appearance, which demonstrated phenotype-environment associations between carapace patterns and habitat at multiple spatial scales^2,8,9,41^. Previous work also showed that crabs from more uniform habitats exhibited less carapace patterning than crabs from heterogeneous habitats. The prominent markings found on some crabs, which tend to arise at the body margins, have also been suggested to provide disruptive camouflage^2^. Our study also ties in with other recent work, showing that with ontogeny, crabs tend to adopt more uniform dark-green appearances, closer to mudflat backgrounds, and that this translates into a survival advantage^42^. Overall, shore crabs therefore appear to utilise strategies that are well suited to the environments in which they live. Next, experiments are needed to test the effectiveness of disruptive edges on the survival (or predator detection) of shore crabs. This is non-trivial, however, since the changing nature of the environment (due to tides, waves, weather) makes experiments based on model prey (e.g. made of Plasticine) challenging to design robust yet visually accurate prey and record predation events, as well as targeting the wide diversity of predatory taxa.

Our work demonstrates how the presence and likely utility of different camouflage markings depends on the visual environment. While this is often discussed^12,18,43^, little work has tested such ideas, especially in natural systems. In uniform environments, a close match to large areas of the background is possible since there is also no ambiguity over the best colour or pattern to match. In low contrast invariable habitats, disruption involving high contrast non-matching markings may actually give an individual away. In contrast, in highly variable backgrounds while it may be possible for different individual phenotypes to match different patches^44^, or for animals to adopt generalist or ‘compromise’ camouflage strategies that match no background perfectly but several to some degree^45,46^, it may sometimes still be better to match the most common substrate^47^. Alternatively, as we demonstrate here, another approach is to focus on using disruptive camouflage, which may provide a better route to concealment irrespective of the specific background patches encountered^18,26^. In addition, the visually complex nature of the rock pool environment likely offers further protection independent of background matching, since high background complexity is known to facilitate camouflage^48,49^.

Disruption is widely considered to work best alongside background matching^30,32^, though there is evidence that to an extent it may also work independently and allow camouflage even with relatively mismatched individuals^21^. Here, we have shown in a natural system, with complex real animal markings and two different background types, that disruption seems to be utilised when background matching is ineffective, and vice versa. Why rock pool crabs had marginally stronger disruptive effects when superimposed onto mudflat backgrounds is unclear, but a possible explanation may be that rock pool crabs seen against homogeneous habitats benefitted from higher levels of differential blending, enabling large parts of the carapace to blend into the background while other aspects stood out^3,18^. Regardless, this finding provides support that disruptive patterning is a distinct camouflage strategy not fully reliant on matching the background^22^.

While rock pool crabs have greater levels of disruption, this may not be the sole utility of their patterns. Diverse markings may contribute to background matching in some instances, and high levels of phenotypic diversity (especially in rock pool habitats^9^) may reduce the ability of predators to detect individuals by hindering the formation of search images^50^. Furthermore, in common with other crustaceans, carapace patterns in shore crab become less distinct with age^8,9,37,39,42,51^. Here, we compared crabs of the same size range and so a reduction in pattern in mudflat crabs cannot be explained by ontogenetic changes. An absence of pattern could in theory also be caused by strong visual predation removing patterned crabs from mudflat environments. However, predation levels are unlikely to be high enough for this, or to override the strong recruitment of new individuals into each location via continuous settlement of post-larvae from their planktonic larval stage and through movement of crabs^8,9,41^. More likely, our results stem from the considerable plasticity in individual shore crab pattern and coloration^35,40,52^. An additional possibility is that individuals show background choice of substrates for concealment, which has been found in a range of other species and taxa^53^, including ghost crabs^54^. Indeed, microhabitat associations in shore crab appearance and substrate have been found in past work that are difficult to explain without some level of background choice existing^8,41^. However, past work directly investigating background choice has focussed on decisions within habitats and often at very fine spatial scales, and here we are considering sites and habitats separated by several km. While shore crabs have been known to move up to 2 km in a short space of time^55^, it seems implausible that they move such distances and among habitats for the purpose of selecting appropriate background environments for camouflage. Nonetheless, this remains an area that would benefit from further research.

Our work highlights the need to understand camouflage strategies more comprehensively in natural systems. A range of previous studies on animal coloration have undertaken comparative analyses, finding evidence for links between camouflage and other defensive strategies with aspects of habitat and life-history (e.g.^56,57^). Disruptive coloration has been suggested to be extremely common in nature^26^, but it is seldom quantified, nor how its characteristics relate to the visual environment in which the bearer is found. Other work, especially on rapid colour changing (in seconds) species such as cuttlefish has explored the expression of camouflage patterns on different visual backgrounds, showing for example that backgrounds of different contrasts and spatial information tend to elicit more uniform or pronounced marking types^31^. One study in particular has also investigated both colour and pattern match of colour-changing flatfish to a range of visual systems, showing that expression of markings is habitat-dependent and that the flounder match the spatial scale of sand and gravel well, but not rocks^58^. This latter finding is interesting because it is consistent with the crabs in our study showing poor match to the rock pool substrates. However, the above study deviates from ours in that it analysed background matching but not disruptive coloration, and did not test how camouflage types/strategies are expected to vary in use with habitat type. In general, more work is needed in this area.

Finally, another key area for further investigation is how phenotype-environment matching links to non-reversible ontogenetic changes in appearance with age and size. In shore crabs, we have also shown that larger crabs progress to a more uniform green coloration as they grow, at least partly independently from the background environment, and that this appears to facilitate generalist camouflage across many habitats, especially when crabs are larger and more mobile^42^. To what extent ontogenetic and plastic changes explain overall phenotypes, and how this relates to camouflage strategy, requires considerable future work. Given that the expression and optimisation of camouflage has been a fruitful area for exploring a variety of issues, from evolutionary outcomes and mechanisms of adaptation (e.g.^59^) through to how visual perception works^60^, testing the presence and efficacy of camouflage features in complex visual scenes in real animals is an important area. This also has the potential to inform on how conspicuous signals improve efficacy in different habitats, from sexual and social signals to aposematism.

## Methods

### Field Sites and Photography

Images were taken of rock pool and mudflat habitats across six sites, three were rock pool habitats (47 background images) and three were mudflats (47 images). Although there was some variation in features among rock pool sites, in general the background substrate was similar, consisting of large clusters of rocks, forming deep gullies filled with small pebbles and sand, alongside small pools (see^9^ for habitat assessments; Fig. 1). Conversely, mudflats consist of large expanses of dark brown mud and surface algae, with little shelter other than dispersed rocks or objects. Sites were located on both north and south coasts of Cornwall, Southwest UK and were separated by between six and 50 km. Gyllyngvase beach (50° 8′ 39.42″N, −5° 4′ 5.244″W) in the Falmouth area, Kennack Sands (50° 0′ 23.695″N, −5° 9′ 28.258″W) located further down the southwest coast, and Perranuthnoe (50° 6′ 43.383″N, −5° 26′ 28.142″W) on the south coast were rock pool sites. For mudflats, Penryn (50° 9′ 49.335″N, −5° 5′ 2.124″W) and Helford (50° 5′ 23.1″N, −5° 9′ 58.754″W) were chosen on the south coast, with Hayle (50° 11′ 36.979″N, −5° 25′ 47.973″W) on the north coast. After entry to the focal area of a site, images were taken separated by approximately 3.7 m with 20 images taken at each site. Some images were deselected back in the lab if they were deemed to be too out of focus, resulting in 47 images overall per habitat. All work was conducted under approval from the University of Exeter ethics committee (application number: 2016/1162).

Image acquisition followed standard protocols^8,9,13^. Images of backgrounds were taken with a Nikon D7000 digital camera modified with a quartz conversion to allow for UV sensitivity (Advanced Camera Services, Norfolk, UK) fitted with a Nikon Nikor 105 mm lens. All photographs were taken in RAW format with fixed aperture settings. The camera was held in position using a tripod and all photographs were taken at the same height (approximately 1 m). Two sets of images were taken, using a visible (Baader UV/IR Cut filter) and UV (Baader Venus U filter) filter, to block UV and infrared light (human-visible images) and allow only UV transmission between 300–400 nm (UV images), respectively. Our camera sensitivities are as follows: UV: 360–400 nm (peak 380 nm), SW: 400–550 nm (peak 460 nm), MW: 420–620 nm (peak 540 nm), LW: 560– 700 nm (peak 625 nm)^61^. To keep light conditions uniform, images were taken on overcast, cloudy days. A photographic umbrella was also used for each photograph to minimise glare, and a black and white reflectance standard with a scale bar was placed in the corner of each image for subsequent image calibration and standardisation^62^. The standard was made from 10 × 10 mm sections of zenith diffuse sintered PTFE sheet (Labsphere, Congleton, UK) and reflected 8.2% and 94.8% of all wavelengths respectively^63^. A scale bar was used to automatically resize all images to the same scale for subsequent pattern and disruption analysis.

For the purposes of this study, we focussed on small juvenile crabs with <15 mm carapace width (CW) at the widest point. While past work has categorised crabs as ‘adults’ when CW > 25 mm, there is a gradual decrease in patterning and a change in the body appearance towards more uniform green as crabs develop^2,8,9^. The reasons for this likely reflect a switch to a generalist camouflage strategy in adults that are more mobile across sites than juveniles^58^. We therefore focus on small juvenile crabs that are much more likely to require habitat-specific camouflage and which are likely to incur greater predation risk than adults^8,34,63^. In total, 97 crabs were sampled and used for the background matching analyses (colour, luminance, and pattern) and 86 of these were used for the disruptive coloration analysis.

Collection of crabs followed past approaches^8,9,52,58^, with sampling at low tide by systematically searching the substrate, lifting seaweed, rocks, and raking the substrate with fingers to locate individuals in a given area. Crabs were then transported back to the laboratory at the University of Exeter, Penryn Campus in clear tanks containing salt water from the habitat and background substrate to cover the bottom of the tank, providing refuge to avoid inflicting stress during transportation. Individuals were then gently dried with tissue paper and placed underneath a tripod set up in a dark photography room. Each crab was placed on a spectrally flat sheet of 2 mm thick black foam with a reflective white PTFE cylinder surrounding the individual to diffuse the light for photography.

### Image analysis and vision modelling

Multispectral images were created using the ‘multispectral image calibration and analysis toolbox’ in Image J^61^. Images were aligned and the white and black standards selected to allow images to be linearised with regards to radiance and standardised to control for light conditions^61,62^. Images were resized downwards to the same scale using the scale bar^9^. Once these images had been calibrated, regions of interest (ROIs) were selected for measurement. Here, the carapace of each crab, excluding appendages, was selected.

We modelled the visual system of both a predatory bird and fish. Among the main predators of shore crabs are shore birds and fish^37,64^. Most birds are probably tetrachromats, using four cone types in colour vision: longwave (LW), mediumwave (MW), shortwave (SW) and ultraviolet/violet (UV/V). Most shore birds have a ‘violet’ cone type relatively more sensitive to longer violet wavelengths than some other more UV-sensitive birds^65^. Therefore, for modelling we followed^8^ and used the visual sensitivity of the peafowl (*Pavo cristatus*)^66^, which is widely used as a species for visual modelling of violet birds (Fig. 1). Although other birds that may be relevant predators (e.g. gulls) can have an ‘ultraviolet’ type system more sensitive to UV light, the crabs and backgrounds in our study generally have low ultraviolet reflectance. For fish, the European pollack, *Pollachius pollachius*^67^ is thought to be a key predator of crabs and represents a dichromatic fish predator, with LW and SW sensitive cones. While fish in our study site can also be trichromats, past work has showed only minor differences in modelled crab appearance among di- and tetrachromatic systems^8^, and so other visual systems are unlikely to vary greatly either. We converted standardized images to predicted cone catch data for each species using a widely implemented polynomial mapping technique^62^. This has been repeatedly shown to provide highly accurate data compared to cone catch modelling with reflectance spectra (see^25,61,68^).

### Background matching: colour and luminance

To quantify colour and luminance match to the background we used a widely employed log version of a model of predator discrimination^69^. This calculates just noticeable differences (JNDs) between two objects to determine discriminability. The output of the model, JNDs, predicts whether two objects can be discriminated (values < 1.00), with increasing values equating to a reduction in the level of camouflage match. For full details see Supplementary Material. We measured the cone values for each crab ROI for each visual system, and then the same for each background image. Using the above models, we then compared the colour and luminance match of each crab carapace to every background image, followed by calculating average colour and luminance JNDs for each crab to each habitat. We therefore derived an average level of background matching for each crab to each of the two habitat types, across all samples.

### Background matching: pattern

To assess background pattern matching between crab carapace and the background for each habitat, a granularity analysis was conducted^9,70,71^ – see Supplementary Material. We used a modification of this process to make direct comparisons between the body markings of an animal and the substrate (‘pattern energy difference’, PED), giving a measure of background pattern matching that predicts detection by wild predators^13^ and humans searching for computer targets^23^. For pattern analysis we used the double cone (luminance) values of the peafowl (*Pavo cristatus*)^66^. Any two patterns with similar energy across all spatial scales will produce low pattern difference values, indicative of background matching, whereas deviation in either amplitude or shape of the spectra will produce larger differences. Here, the absolute difference between the spectra of crab carapaces and habitat backgrounds was assessed (both rock pool and mudflat separately). As above, we derived an average level of pattern matching for each crab to each of the two habitat types.

### Disruptive coloration

To quantify edge disruption, we used a recently developed method called ‘GabRat’, which uses angle sensitive filters to measure the ratio of false edges to coherent edges around the target outline^23^ – see Supplementary Material. A high ratio of false edges to coherent edges should be more disruptive, and therefore indicates that prey are more difficult to detect, while lower values suggest salient coherent edges. While we note that GabRat is relatively new and awaits greater testing, especially in natural systems, the metric has been shown to be one of the most important predictors of human detection times of disruptive targets (and superior to other pattern metrics, including those for quantifying disruption based on more conventional edge detection algorithms^23^). For each image, each crab was randomly placed in 50 different positions that did not overlap with each other or any exclusion zones. This was repeated on all 94 backgrounds (47 rock pool and 47 mudflat), resulting in a total of 4700 edge disruption measurements per individual crab. This process accounted for variation in positioning of crabs in the wild. The average GabRat value of the total 50 positions was calculated for each background, so that one value was generated per crab/image combination. Means per individual were then calculated across both rock pool and mudflat backgrounds, so that each crab had an average edge disruption value for both habitat types.

### Statistics

All individuals (from the two habitat types) were placed onto both habitat types, resulting in two mean values per individual. A split plot 2 × 2 repeated measures mixed factorial ANOVA with type III sums of squares was used to assess the match of individuals to rock pool and mudflat images using the R function *ezANOVA*. Our within subjects factor was image background and the between subjects factor was the collection habitat. Full models including the 2-way interaction were run for each of our dependent variables (the metrics of camouflage): edge disruption data, PED, and colour and luminance data for both avian and fish vision models; so the general form was: (camouflage metric ~ *collection*.*site* + *background*.*habitat* + *collection*.*site* * *background*.*habitat*).

Homogeneity of variance was assessed using Levene’s test. Colour JND data for avian vision was non-normal and so a log transformation was applied. Assumptions of normality and homogeneity of variance were met in all analyses, which were conducted in the statistical program R^72^.

We predict that crabs from rock pool habitat sites will have higher GabRat edge disruption values than crabs from mudflat habitats. There should also be a habitat effect, with the variation in rock pool backgrounds allowing greater disruption regardless of the crab origins. Conversely, for background matching, crabs from mudflat habitats may better match the background for each metric owing to its more simple nature than crabs from rock pool habitats. In addition, mudflat backgrounds may also allow greater background matching owing to their more uniform appearance, whereas rock pools present highly variable environments meaning matching many patches is not possible.

## Supplementary information

Supplementary Methods

Supplementary Dataset 1

## Data Availability

All data for this study are included as a Supplementary File.
