# Supplementary material for: Background matching and disruptive coloration as habitat-specific strategies for camouflage
Source: Sci Rep. 2019 May 24;9:7840. doi: 10.1038/s41598-019-44349-2 (PMC6534618; doi:10.1038/s41598-019-44349-2)
Supplement: Supplementary file 1 — Supplementary Methods [file 41598_2019_44349_MOESM1_ESM.pdf]

Supplementary Material

Background matching and disruptive coloration as habitat-specific strategies for camouflage

Natasha Price[1], Samuel Green[1], Jolyon Troscianko[1], Tom Tregenza[1], Martin Stevens[1]*

Centre for Ecology and Conservation, College of Life and Environmental Sciences, University of Exeter, Penryn Campus, Penryn TR10 9FE, UK

***Correspondence:** martin.stevens@exeter.ac.uk

This file contains the following supplementary information

Further methods for calculating camouflage metrics

Calculating Metrics of Camouflage

Background matching: colour and luminance

To quantify colour and luminance match to the background we used a widely employed log version of a model of predator discrimination[1]. This calculates just noticeable differences (JNDs) between two objects to determine discriminability. Based on a recent evaluation of estimates of receptor noise[2], we used Weber fractions of 0.1 and 0.05 for the bird and fish vision model respectively, and cone proportions of LW = 0.92, MW = 1.00, SW = 0.81, UV = 0.54 for the peafowl[3] and LW = 1.0 and SW = 0.5 for the pollack[4]. For luminance, we used a modified achromatic version of the model[5] based on the double cones for birds and LW cones for the pollack[6], with Weber fractions of 0.2 and 0.05 for the bird and fish models[2]. The output of the model, JNDs, predicts whether two objects can

be discriminated (values < 1.00), with increasing values equating to a reduction in the level of camouflage match. We measured the cone values for each crab ROI for each visual system, and then the same for each background image. Using the above models, we then compared the colour and luminance match of each crab carapace to every background image, followed by calculating average colour and luminance JNDs for each crab to each habitat. We therefore derived an average level of background matching for each crab to each of the two habitat types, across all samples.

Background matching: pattern

To assess background pattern matching between crab carapace and the background for each habitat, a granularity analysis was conducted. This method has previously been successfully used to analyse patterns in other animals, including cuttlefish (e.g.[7,8]) and cuckoo-host bird eggs (e.g.[9,10]). It has also been used to measure shore crab carapace markings[11,12]. During granularity analysis, each image is filtered using Fast Fourier bandpass filtering, at multiple spatial frequency scales, resulting in each filter measuring information or 'energy' at different spatial bands (see[7,9]). The energy at each of these scales can be measured as the standard deviation of the pixel values[13], such that larger markings (of low spatial frequency) are captured by smaller filter sizes, and smaller markings (higher spatial frequency) are captured by larger filter sizes. While granularity analyses of intrinsic animal patterns can provide valuable measures of appearance, the process has recently been extended to allow direct comparisons between the body markings of an animal and the substrate ('pattern energy difference', PED), giving a measure of background pattern matching. This has been used to measure background matching in birds[14] and fish[15], and has been shown to predict detection times of predators searching for wild bird nests (e.g. predict survival[16]) and of humans searching for hidden computer targets[17].

While colour is important in many detection and discrimination tasks, achromatic vision is widely thought to be key for analysing much spatial information, especially pattern, and in birds this seems based on the double cones[6]. Therefore, for pattern analysis we used the double cone

(luminance) values of the peafowl (*Pavo cristatus*)[3]. Here, because previous work has shown close similarities between avian and fish crab pattern achromatic metrics[18], and because achromatic information tends to be more conserved among species and based on middle to longer-wave parts of the spectrum[6], we only model avian and not fish based pattern measures. PED values were generated in ImageJ as part of a granularity analysis, which calculates the absolute difference between the spectra of two images/samples across all spatial scales measured. Any two patterns with similar energy across all spatial scales will produce low pattern difference values, indicative of background matching, whereas deviation in either amplitude or shape of the spectra will produce larger differences. Here, the absolute difference between the spectra of crab carapaces and habitat backgrounds was assessed (both rock pool and mudflat separately). For each background image, we measured the granularity spectrum of the entire image. The granularity spectrum of each crab carapace was then compared to the spectrum of every background image, and the average per habitat taken. As above, we therefore derived an average level of pattern matching for each crab to each of the two habitat types.

Disruptive coloration

To quantify disruption, we used a recently developed method inspired by visual processing that has been shown to be one of the most important predictors of human detection times of disruptive targets (and superior to other pattern metrics[17]). This method, unlike other metrics of disruption (e.g.[19,20]), takes account the direction of perceived edges versus actual body outlines, enabling it to distinguish ‘false edges’ (markings that run at right angles to the prey’s outline and are maximally disruptive) from ‘coherent edges’ (markings that match the outline of the animals body, potentially making the prey’s shape easier to detect). The resultant edge disruption metric, ‘GabRat’, uses angle sensitive filters to measure the ratio of false edges to coherent edges around the target outline[23]. A high ratio of false edges to coherent edges should be more disruptive, and therefore indicates that prey are more difficult to detect, while lower values suggest salient coherent edges.

First, images were exported as binary mask images (the crab carapace being white against a black background) in TIFF format, and a Gabor filter was applied to each of the pixels around the edge of the carapace, using a sigma level (filter size) of 5 as this was most relevant for objects around 5-15 mm[17]. The filter size is dependent on the px/mm of the carapace and controls the size of the pattern markings that are detected; larger sigma levels would detect larger disruptive patterns. The edge disruption of each crab was in this way measured against a neutral background. This enabled the angle of the crab's outline at each point of the carapace to be measured independent of the background, since the metric involves comparing the presence of edges perpendicular to, and intersecting the body outline. This process was then repeated, but for the actual crab when against all rock pool and mudflat image backgrounds, using the same sigma level. A custom function in ImageJ randomly placed each crab onto a background and measured the carapace edge disruption by applying the Gabor filter. This measured each point around the crab's outline at an angle parallel to and at right angles to the true outline, enabling measurements of the interaction between the crab and its background to be made. An aspect of image analysis of the background samples was to ensure that only relevant areas of substrate for potential camouflage were selected. As such, any possible areas that a crab would not normally be found on in the wild (at least during the day; e.g. sitting on top of large rocks), were labelled exclusion zones in the image processing and ignored. To avoid ambiguity, we selected exclusion zones as any substrate object (e.g. exposed rock) that had a width larger than 7.5 mm. A disruption ratio was calculated at each point on the crab's outline, and the mean of these ratios across the whole carapace outline was calculated, resulting in the final Gabor edge disruption ratio (GabRat). GabRat values range from 0 - 1, with < 0.2 being considered as equating to a low level of edge disruption and > 0.4 to high edge disruption[17].

For each image, each crab was randomly placed in 50 different positions that did not overlap with each other or any exclusion zones. This was repeated on all 94 backgrounds (47 rock pool and 47 mudflat), resulting in a total of 4700 edge disruption measurements per individual crab. This process accounted for variation in positioning of crabs in the wild. The average GabRat value of the

total 50 positions was calculated for each background, so that one value was generated per crab/image combination. Means per individual were then calculated across both rock pool and mudflat backgrounds, so that each crab had an average edge disruption value for both habitat types.

References

1. Vorobyev, M. & Osorio, D. Receptor noise as a determinant of colour thresholds. *Proc R Soc B* **265**, 351-358 (1998).
2. Olsson, P., Lind, J. & Kelber, A. Chromatic and achromatic vision: parameter choice and limitations for reliable model predictions. *Behav Ecol* **29**, 273-282 (2018).
3. Hart, N. S. Vision in the peafowl (Aves: *Pavo cristatus*). *J Exp Biol* **205**, 3925-3935 (2002).
4. Shand, J., Partridge, J. C., Acher, S. N., Potts, G. W. & Lythgoe, J. N. Spectral absorbance changes in the violet/blue sensitive cones of the juvenile pollack, *Pollachius pollachius*. *J Comp Physiol A* **163**, 699–703 (1988).
5. Siddiqi, A., Cronin, T. W., Loew, E. R., Vorobyev, M. & Summers, K. Interspecific and intraspecific views of color signals in the strawberry poison frog *Dendrobates pumilio*. *J Exp Biol* **207**, 2471-2485 (2004).
6. Osorio, D. & Vorobyev, M. Photoreceptor spectral sensitivities in terrestrial animals: adaptations for luminance and colour vision. *Proc R Soc B* **272**, 1745-1752 (2005).
7. Chiao, C.-C., Chubb, C., Buresch, K. C., Siemann, L. & Hanlon, R. T. The scaling effects of substrate texture on camouflage patterning in cuttlefish. *Vis Res* **49**, 1647-1656 (2009).
8. Barbosa, A. *et al.* Cuttlefish camouflage: the effects of substrate contrast and size in evoking uniform, mottle or disruptive body patterns. *Vis Res* **48**, 1242-1253 (2008).
9. Stoddard, M. C. & Stevens, M. Pattern mimicry of host eggs by the common cuckoo, as seen through a bird's eye. *Proc R Soc B* **277**, 1387-1393 (2010).
10. Stevens, M., Troscianko, J. & Spottiswoode, C. N. Repeated targeting of the same hosts by a brood parasite compromises host egg rejection. *Nat Comm* **4**, 2475 (2013).

11. Nokelainen, O., Hubbard, N., Lown, A. E., Wood, L. E. & Stevens, M. Through predators' eyes – phenotype-environment associations in shore crab coloration at different spatial scales. *Biol J Linn Soc* **122**, 738–751 (2017).

12. Stevens, M., Wood, L. E. & Lown, A. E. Camouflage and individual variation in shore crabs (*Carcinus maenas*) from different habitats. *PLoS ONE* **9**, e115586 (2014).

13. Troscianko, J. & Stevens, M. Image calibration and analysis toolbox – a free software suite for objectively measuring reflectance, colour and pattern. *Meth Ecol Evol* **6**, 1320–1331 (2015).

14. Stevens, M., Troscianko, J., Wilson-Aggarwal, J. & Spottiswoode, C. N. Improvement of individual camouflage through background choice in ground-nesting birds. *Nat Ecol Evol* **1**, 1325–1333 (2017).

15. Smithers, S. P., Wilson, A. & Stevens, M. Rock pool gobies change their body pattern in response to background features. *Biol J Linn Soc* **121**, 109–121 (2017).

16. Troscianko, J., Wilson-Aggarwal, J., Stevens, M. & Spottiswoode, C. N. Camouflage predicts survival in ground-nesting birds. *Sci Rep* **6**, 19966 (2016).

17. Troscianko, J., Skelhorn, J. & Stevens, M. Quantifying camouflage: how to predict detectability from appearance. *BMC Evol Biol* **17**, 7 (2017).

18. Nokelainen, O., Hubbard, N., Lown, A. E., Wood, L. E. & Stevens, M. Through predators' eyes – phenotype-environment associations in shore crab coloration at different spatial scales. *Biol J Linn Soc* **122**, 738–751 (2017).

19. Stevens, M. & Cuthill, I. C. Disruptive coloration, crypsis and edge detection in early visual processing. *Proc R Soc B* **273**, 2141-2147 (2006).

20. Lovell, P. G., Ruxton, G. D., Langridge, K. V. & Spencer, K. A. Individual quail select egg-laying substrate providing optimal camouflage for their egg phenotype. *Curr Biol* **23**, 260-264 (2013).
